# Automated segmentation of the median nerve in patients with carpal tunnel syndrome

**DOI:** 10.1038/s41598-024-65840-5

**Published:** 2024-07-20

**Authors:** Florentin Moser, Sébastien Muller, Torgrim Lie, Thomas Langø, Mari Hoff

**Affiliations:** 1 Department of Rheumatology, St. Olavs Hospital, Trondheim, Norway; 2 Department of Neuromedicine and Movement Science, Norwegian University of Science and Technology, Trondheim, Norway; 3 Department of Health Research, SINTEF Digital, Trondheim, Norway; 4 Department of Research, St. Olavs Hospital, Trondheim, Norway

**Keywords:** Image processing, Musculoskeletal system

## Abstract

Machine learning and deep learning are novel methods which are revolutionizing medical imaging. In our study we trained an algorithm with a U-Net shaped network to recognize ultrasound images of the median nerve in the complete distal half of the forearm and to measure the cross-sectional area at the inlet of the carpal tunnel. Images of 25 patient hands with carpal tunnel syndrome (CTS) and 26 healthy controls were recorded on a video loop covering 15 cm of the distal forearm and 2355 images were manually segmented. We found an average Dice score of 0.76 between manual and automated segmentation of the median nerve in its complete course, while the measurement of the cross-sectional area at the carpal tunnel inlet resulted in a 10.9% difference between manually and automated measurements. We regard this technology as a suitable device for verifying the diagnosis of CTS.

## Introduction

Median nerve entrapment at the carpal tunnel is the most common peripheral nerve compression syndrome and causes substantial morbidity and economic burden^1,2^. Many different causes have been identified which include intrinsic factors like obesity, diabetes or pregnancy, as well as occupational factors like repetitive work or use of vibrating machines. However, in many cases the cause is elusive^2^. The reported prevalence of carpal tunnel syndrome (CTS) varies depending on method and definition. Atroshi found a prevalence of 2.7% in southern Sweden based on self-reported symptoms verified by clinical examination and a nerve conduction study^3^, while Ferry estimated a prevalence between 7 and 16% in the United Kingdom^4^. Females are found to have CTS more often than men and the median age at the time of diagnosis is about 50 years^5^.

Diagnosis is usually based on clinical examination and additional nerve conduction^6,7^ or imaging studies^5^. Ultrasound is a method that can be used to verify the diagnosis of CTS^8^. Features of the median nerve (MN) entrapment include flattening of the nerve^9^, increased stiffness, intraneural vascularity or venous congestion^10–12^, decreased mobility, change in echogenicity of the nerve^13^ and increased cross-sectional area. Increased cross-sectional area is the most validated of these parameters^14^.

In contrast to nerve conduction studies, ultrasound not only visualizes the swollen nerve, but also can reveal local pathologies that may affect the nerve as tumors, ganglions or tendinopathy. A general drawback of ultrasound is its operator dependency, therefore substantial effort has been put into formalizing and standardizing ultrasound studies in rheumatology^15,16^. Additional technologies such as automated annotation, segmentation, and measurement of critical structures by machine learning algorithms have been proposed to minimize operator-dependency^17–19^.

Two topics of debate in CTS are the optimal site of measurement of the cross-sectional area (CSA) of the median nerve and the cut-off used to determine whether the nerve is pathologically enlarged. This is because of the relatively wide range of CSA depending on sex, age, site of measurement, ethnicity and method of measurement. One review reported a CSA at the middle-forearm and at the carpal tunnel inlet of 7.07 mm^2^ and 8.27 mm^2^, respectively, with generally larger CSA in men compared to women, possibly more related to higher weight^20^. Some authors propose measuring CSA at the entrance of the carpal tunnel, while others recommend measuring inside the carpal tunnel or comparing CSA at different locations like proximal, inside or distal of the carpal tunnel, respectively^21^.

The scope of this paper comprises segmentation of the median nerve covering its course along the distal half of the forearm and comparing manual measurements with automated measured CSA. This includes measurements of the CSA at the carpal tunnel inlet, comparing the manual measurement of CSA with automated measured CSA, measurement of CSA by two operators as well as comparing the inter-observer variability with the variability between manual and machine measurements.

## Methods

### Subject inclusion

Patients with CTS were recruited from the orthopedic department St. Olavs hospital, Trondheim (Norway). All patients scheduled for surgery with carpal tunnel release were defined as established CTS. Exclusion criteria were previous fracture, previous CTS operation, and patients with established inflammatory arthritis. The result from the ultrasound exam did not interfere with the decision to treat by surgical release. Patients waiting for the operation were asked to do the ultrasound before the operation. The healthy controls were recruited from the rheumatologic department and SINTEF, the exclusion criteria were symptoms of CTS, former surgery or severe trauma of the wrist. Approval by the Regional Committees for Medical and Health Research Ethics (*Regionale komiteer for medisinsk og helsefaglig forskningsetikk* in Norwegian) was granted based on detailed consideration of our experimental protocol (application ID 373038). The research was performed in accordance with national and EU guidelines and regulations, written informed consent was obtained from all participants, and our research was performed in accordance with the WMA Declaration of Helsinki—Ethical principles for medical research involving human subjects.

Ground truth or gold standard for the diagnosis of CTS: The diagnosis of CTS was defined as established when the patient was accepted for surgery. But not all patients were diagnosed with electrophysiological tests, in some cases the patient was accepted for surgery only after a clinical evaluation and a history of a successful decompression of the contralateral hand.

From all participants, age, sex, self-reported weight and height were recorded. For the ultrasound scanning the participant was sitting in front of the operator with the arm in a supine position on a table with a slight flexion of the fingers. From the wrist crease was measured a distance of 15 cm up the forearm where ultrasound was taken. The reason to scan such a large aspect of the median nerve was to secure enough data for future studies on 3D visualisation of the nerve and for the possibility to calculate ratios of the CSA at different levels.

From the healthy controls only the left forearm and wrist were scanned with ultrasound. From the patients only the arm that was planned for operation was scanned (in patients with bilateral CTS both hands were scanned). All ultrasound images were taken by a rheumatologist with more than 5 years of experience in musculoskeletal ultrasound (F.M.) To guarantee visualization of the median nerve in the middle of the forearm, a GE Logiq 10 scanner was used with a 6–15 MHz probe in a MSK mode with a depth of 3.5–4.0 cm was used. Three similar recordings were taken from each participant. Each video-loop of the ultrasound consisted of about 500 frames, depending on how fast the probe was moved down the forearm.

### Segmentation

**Figure 1:**
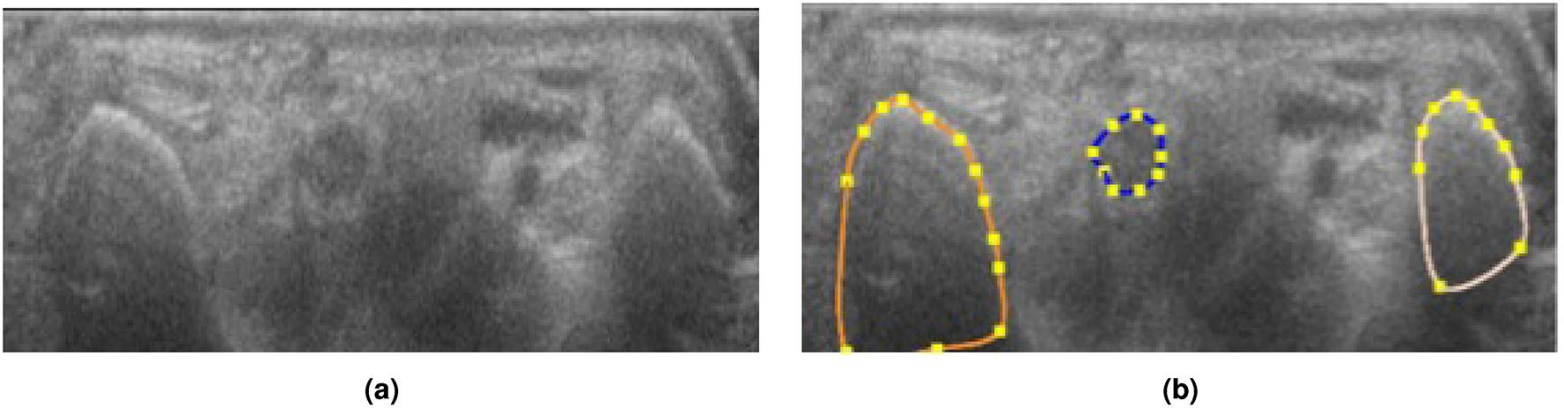
(**a**) Wrist flexor side, transverse at the crease without segmentation, (**b**) same frame as in A, but outlined the scaphoid bone to the left (orange), the pisiform on the right side (white), and the median nerve (blue).

The ultrasound videos were manually segmented by an experienced rheumatologist using Annotation Web^22^ to delineate the median nerve by a polygon in 30–40 ultrasound images per participant. Each anatomical structure that was segmented, a different colour was assigned to (Fig. 1b). The epineurium (nerve sheath) of the median nerve was not included in the segmentation or outlining (Fig. 1). The images selected for segmentation were selected from all parts of the ultrasound scans, not only close to the wrist. Images were chosen according to their quality, so in areas with very blurry images segmentation was dropped, but in areas with good quality in the sense of distinguishability of the structures, images were segmented in close succession.

### Measurement of the median nerve

For the measurement of the cross-sectional area (CSA) of the median nerve, only images taken to the very inlet of the carpal tunnel with visualization of both the scaphoid and the pisiform were used. The CSA of the median nerve was manually traced at the inside of the high echogenic epineurium from each participant. We performed three manual measurements from each participant and copied the CSA calculated by the scanner. Additionally, a similar measurement was taken further proximally at the level of the pronator quadratus muscle. The trained AI algorithm was used to automatically calculate the CSA from the same image that were used for the first manual CSA measurement for each patient.

### Inter-observer variability

In a minor sample of the patients (n = 8), manual segmentation of the median nerve was performed by F.M. and I.S. separately for inter-observer testing. From each participant 30 frames were randomly picked by a non-expert, so both operators were annotating identical frames.

### Algorithm training

The images used to train the segmentation AI-models were exported from the GE Logic scanner with minimal noise filtering and processing. The images were downscaled to fit a network input size of 256 $\times$ 256 pixels. For the algorithm training the ultrasound images were used in their unprocessed form without any augmentation of contrast The CNN architecture used in this study was a fully-convolutional encoder–decoder U-net type network^23^. This architecture has six levels with cross-over connections and uses 2 $\times$ 2 max pooling in the encoder and 2 $\times$ 2 repeat upsampling in the decoder. Two 3 $\times$ 3 convolution layers are used at each level, together with ReLU activation. The output is a segmentation of the same size as the input image.

The neural networks were trained using Keras with 10-fold cross-validation, Adam optimizer, 150 epochs and a Dice loss function. The patients were randomly divided into 10 groups with 4–5 patients in each group. For each of the cross-validation folds, data from one group was set aside for testing of the trained model, one group was used as a validation set during training and 8 groups were used as a training set. The test results from each fold were averaged for the final test result. Random augmentations were used during training to reduce overfitting^19^. The following augmentations were used:

- Gamma intensity transformation.
- Rotation with a maximum angle of 10 degrees.
- Gaussian shadows: Dark shadows applied to the image at random locations and with random sizes.
- Depth: Cuts the image bottom at random depths.
- JPEG compression: Compresses the image with a random quality setting.
- Elastic image deformation.

### Diagnostic power of cross-sectional area

A secondary branch of our study, independently from machine learning-based segmentation of the median nerve, was to assess the relationship between the cross-sectional area (CSA) of the median nerve and the patient being diagnosed with carpal tunnel syndrome. This was done using the CSA measured manually by delineating the nerve using a dedicated function of the ultrasound scanner, and a second time using the CSA predicted by the machine learning model to compare if the diagnostic power was comparable to that of the first method.

#### Manually measured cross-sectional area

The data was preprocessed by averaging the three repeated measurements and removing outliers of cross-sectional area (CSA) of the median nerve, following the criteria of 1.5 times interquartile range. Variables were normalized using a standard scaler, to achieve a mean of zero, and standard deviation of one. A preliminary linear regression was fitted to the CSA as a function of height, weight, age, and gender. Variables were further binned to improve parsimony of the following models. A logistic regression was fitted to the diagnostic status of the subject as dependent variable, using a generalized linear model with logit link. All analyses were performed using R Statistical Software (v4.3.3; R Core Team 2024). Independent variables, or predictors, were the manually measured CSAs of the median nerve, as well as height, weight, age, and gender of the patients. The model was weighted to compensate for any uneven distribution of patients versus control. The model was built incrementally from a null model to a set of independent variables so that the marginal goodness of fit overcomes the additional degrees of freedom measured by maximum likelihood and chi squared test.

$$\begin{aligned} Diagnosis \sim CSA_s + height_s \end{aligned}$$

where *Diagnosis* is the binary dependent variable of the logistic regression, and $CSA_s$ and $height_s$ are the scaled or normalised version of CSA of the median nerve and subject height, respectively.

Though this method balances goodness of fit and parsimony, we combined the predictive value of the model for final selection of the model. The latter was calculated by letting the logistic regression predict the classes (standard probability cut-off of 0.5) and building a confusion matrix from results and reference classes of diagnosis. Additional standard metrics of accuracy, sensitivity and specificity are also reported. Reproducibility of the three repeated measurements was tested using the test-retest reliability (intraclass correlation coefficient—ICC).

#### Machine learning-estimated cross-sectional area

The machine learning-simulated cross-sectional areas were compared to the manually measured ones in paired t-test and Wilcoxon paired samples test to determine systematic bias of estimation. Additionally, the diagnostic value of the simulated CSAs was tested using the same logistic regression as for the manual ones. The classification as healthy or carpal tunnel syndrome with the standard metrics allows for comparison of diagnostic powers.

## Results

20 patients and 26 healthy controls were enrolled. Table 1 shows patient and healthy controls characteristics.

**Table 1:** Demographics of the included subjects.

	Number	Gender % (%)	Age (yr)	Weight (kg)	Height (cm)
Healthy controls	16 female	61	45.1 (22–63)	69.2 (45–91)	168 (153–180)
	10 male	39	43.7 (29–53)	88.3 (67–109)	181 (172–188)
Patients	10 female	50	48.3 (35–70)	75.3 (58–93)	166 (156–174)
	12 hands				
	10 male	50	57.9 (27–73)	91.7 (73–120)	178 (168–185)
	13 hands				

Age, weight, and height are reported as mean (range).

In total 2355 images were manually segmented and used for training segmentation AI-models. We trained tenfold cross validation, the results showed an average dice score between manual segmentation and AI-based segmentation of 0.76, with a standard deviation of 0.068. The average precision was 0.84 and average recall was slightly lower at 0.71, indicating that the models had a slight tendency to underestimate the median nerve CSA. The median nerve CSA in healthy subjects was 7.67 mm^2^, while it was 11.7 mm^2^ in CTS patients. The difference is significant with Wilcoxon $P<10^{-4}$, and t-test $P<10^{-5}$.

### Inter-observer variability

**Figure 2:**
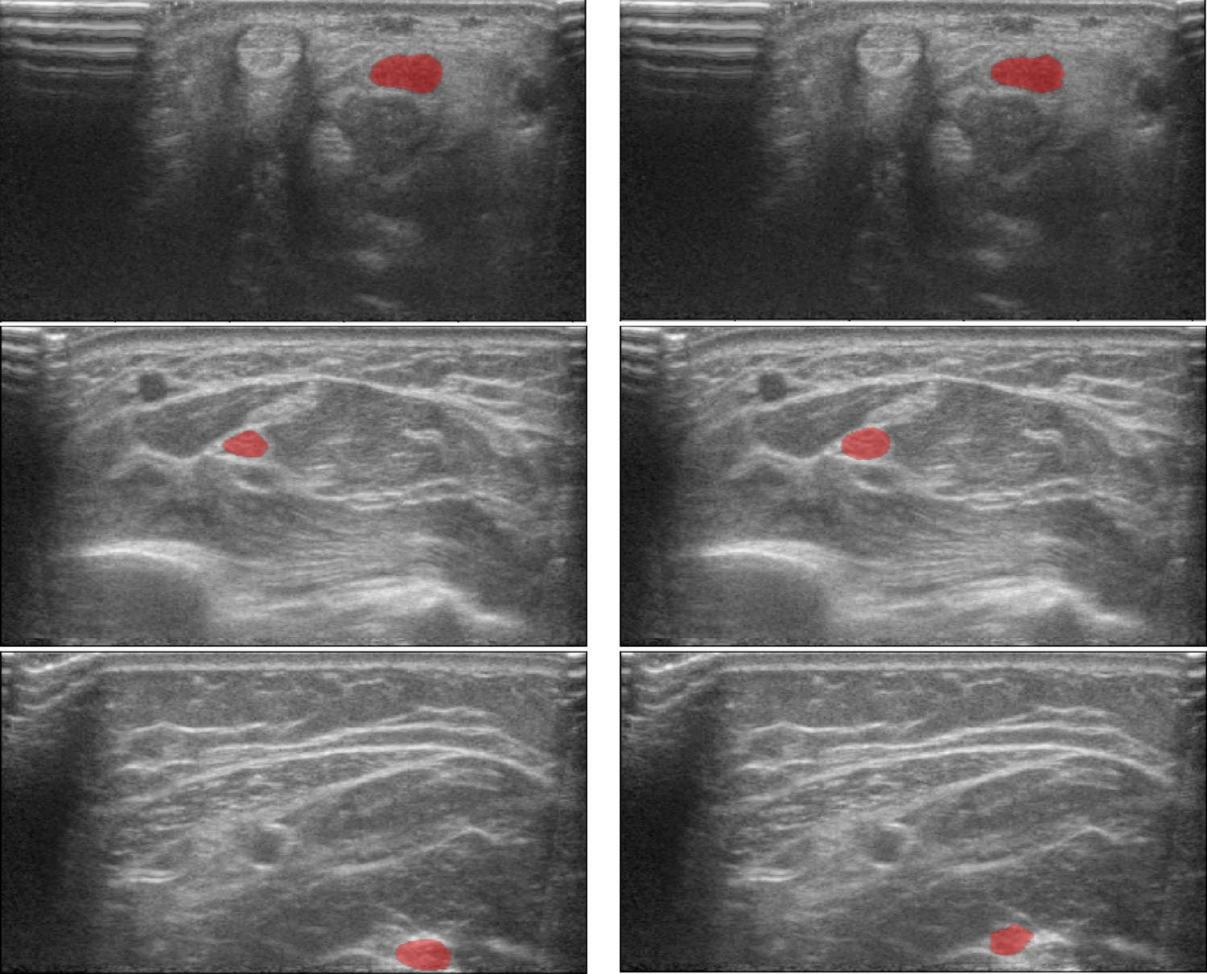
Comparing expert A and B. The left side shows segmentation by expert A, while right shows segmentation of expert B. Top row close to the carpal tunnel inlet, middle row at the level of the pronator quadratus muscle and the lower row in the middle of the forearm. Best agreement is shown in the top row (Dice = 0.94), median in second row (Dice = 0.82), and worst agreement at the bottom (Dice = 0.07). At the left margin of all images some reverberation artefacts.

For the inter-observer variability test, the total number of segmented images (including ‘no segmentation/annotation’) was 246 for seven different subjects. Of these images, only 178 had overlapping delineations. For the overlapping segmentations, the average dice value was 0.78 (Fig. 2). Five images were segmented by both operators, but delineation did not overlap. 45 images were only segmented by expert A, four images were only segmented by expert B and 14 images were not segmented by any of the experts.

### Anatomical variation

Bifid median nerve was found in three out of 51 wrists (5.8%), all healthy subjects. Persistent median artery was found in two cases (both together with a bifid median nerve, an example shown in Fig. 5).

### Measurements of cross-sectional area

**Figure 3:**
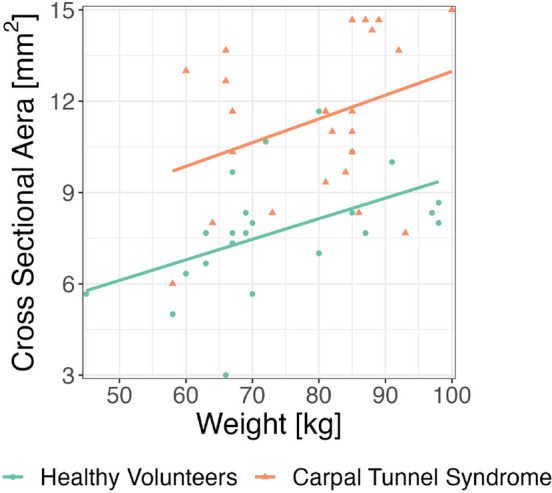
Cross-sectional area of median nerve by body weight.

**Figure 4:**
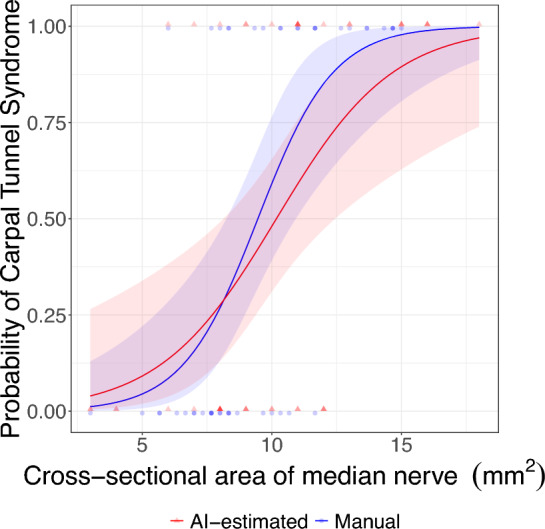
Diagnostic power of the cross-sectional area of the median nerve and 95% confidence interval for manually assessed or AI-estimated measurement. The two logistic regressions provide slightly different cut-offs (at 50% probability) reflecting the bias in the AI-estimation. The observation points are artificially shifted for readability.

The three repeated manual measurements had an intraclass correlation coefficient (ICC) of 0.902 [0.847–941], indicating high reliability of the repeated measurements. The variation of CSA by weight and diagnostic status is shown in Fig. 3. Out of 49 valid entries, 2 cross-sectional areas (CSAs) of the median nerve were removed as outliers. The optimum cut-off point for prediction of CTS, based on maximizing the sum of sensitivity and specificity, was of 9.3 mm^2^. The preliminary linear regression found a significant linear relationship between CSA and weight ($P<0.01$) with medium effect size (standardized slope of 0.458). For the logistic regression (Fig. 4), division into 6 bins was optimal and provided 7 or 8 observations per bin. A model including standardized height and CSA yielded optimal goodness of fit and classification accuracy, though the height variable did not achieve significance in the logistic regression.

**Table 2:** Confusion matrix of the logistic regression for manually measured cross-sectional area of the medianus nerve.

	Reference diagnosis	
Predicted diagnosis	Negative	Carpal tunnel syndrome
Negative	20	5
Carpal tunnel syndrome	4	18

Accuracy (CI): 0.81 (0.67, 0.91), Sensitivity (recall): 0.78, Positive predictive value (precision): 0.82 (Table 2), and an area under the curve (AUC) of 0.89.

Additional measurements of the median nerve CSA at the proximal site (over the pronator quadratus muscle) showed no difference by diagnostic status (unpaired Wilcoxon $P>0.2$, unpaired t-test $P>0.3$), providing no classification information, with an accuracy of 0.51 equal to the no information rate. The ratio of distal-to-proximal CSA provided slightly weaker classification power than distal CSA alone, with an accuracy of 0.79 and (0.64, 0.89) confidence interval.

For the automated measurement the algorithm failed in two cases to find the median nerve. In one additional case, the nerve was bifid and only one branch was detected. These three cases were removed before further analyses to avoid introducing a strong quantitative bias while the issue is categorical. The optimum cut-off for prediction of CTS, point based on maximizing the sum of sensitivity and specificity, was of 11 mm^2^. The CSA estimated by machine learning was not significantly different from the manually measured ones (Wilcoxon paired, $P>0.05$, paired t-test $P>0.1$), with a mean difference of 0.26 mm^2^, a median absolute difference of 1.33 mm^2^, and a mean absolute difference of 1.56 mm^2^. This corresponds to a median absolute relative difference of 10.9% between manually measured and AI-estimated CSA.

**Table 3:** Confusion matrix of the logistic regression for AI-estimated cross-sectional area of the medianus nerve.

	Reference diagnosis	
Predicted diagnosis	Negative	Carpal tunnel syndrome
Negative	17	6
Carpal tunnel syndrome	5	16

The logistic regression for the AI-estimated CSA shows that AI-estimated CSA is a significant predictor of CTS ($P<0.005$). The classification accuracy is 0.75 (0.60–0.87), with a sensitivity (recall) of 0.73, positive predictive value (precision) of 0.76 (Table 3), and an area under the curve of 0.77.

## Discussion

The main finding of this study was that CSA estimated by machine learning was not significantly different from the manually measured ones. Another focus of this study was automated identification of the median nerve along the distant half of the forearm and automated measurement of its cross-sectional area at the inlet of the carpal tunnel. While the automated CSA measurement has been assessed by a number of groups^24^, the automated segmentation of the nerve over a larger area has been less studied.

In total, we achieved a dice score of 0.76 on the automated segmentation of the median nerve along the distal halve of the forearm, which was close to the inter-observer variability at 0.78. To our knowledge, no other study group has published results for automated segmentation of the median nerve as a whole. So far, measurements have been taken around the carpal tunnel or at distinct locations like the pronator quadratus muscle^25^, but not in a continuum.

In our study we segmented the median nerve over a length of 15 cm on the distal forearm. In the middle forearm the nerve is relatively easily identified between the superficial and the deep flexor muscles, but there are two segments where the nerve is more difficult to distinguish from the surroundings: (1) distal to the pronator quadratus muscle where the nerve winds up from the deep to its more superficial localization right before the wrist crease and (2) at the very inlet of the carpal tunnel, when the nerve submerges under the transverse ligament. This may influence the precision. Other reasons for the moderate precision, recall, and dice score, might be caused by our choice to use unprocessed images for algorithm training. This leaves the operator with images with less contrast and less distinguishable structures. Processed images with enhanced contrast render the anatomic structures more distinguishable and makes them better suited for manual segmentation. On the other hand, there is the possibility that machine learning can recognize features in the unprocessed images that cannot be visually appreciated as shown by Faeghi et al.^26^.

When it comes to the manual measurements of the cross-sectional area at the carpal tunnel inlet, our results are very close to others. Dejaco^25^examined 135 patients with suspected CTS and 26 healthy controls, where patients with typical clinical findings and repeated pathological electrophysiological testing were considered as having CTS with over 90% probability. In these patients they found a median CSA at the carpal tunnel inlet of 11.5 mm^2^ (7–28) and 8.0 (6–13) for the controls. These findings are almost identical with ours (11.7 and 7.7 mm^2^ respectively).

Our study group made additional manual measurements at multiple anatomic sites of the distal forearm, but calculating ratios of the CSA at different sites did not translate into higher diagnostic accuracy. Klauser et al.^21^ proposed the use of the difference between CSA measured at the crease of the wrist and the CSA more proximal at the level of the pronator quadratus muscle, but we could not reproduce any additional improvement for diagnostic precision. Torres Costoso et al.^14^ concluded the same way in their thorough review including 28 studies, that the CSA measurement of the median nerve at the carpal tunnel inlet has the highest diagnostic accuracy with no additional value in adding measurements at the carpal tunnel outlet.

For the interobserver variability testing, images were randomly picked distributed along the distant half of the forearm, similar to the segmentation used to train the AI-models. The resulting assortment of images were of very different quality, some of optimal quality and others out of focus. Fourteen images were not segmented by any of the two operators (5.7%), these were images of so poor quality one could not identify the median nerve at all, especially 2–3 cm proximal to the wrist crease, where the median nerve comes up from the deep to its more superficial location close to the carpal tunnel. In images taken at the wrist crease, the median nerve is much better distinguishable. Figure 2 shows in the top row images with almost perfect identical segmentation, but also an example where the two operators segmented two different structures with no overlap at all. The average dice score between the two annotators for the images where the delineations did overlap, were very similar to the results for the automatic segmentation.

As earlier mentioned, the CSA is not the only feature of the median nerve that alters under compression, others are the nerves echogenicity and vascularity^27^. These alterations can be exploited by machine learning as newly described by Shinohara et al.^28^, who report an impressing diagnostic accuracy of 0.96 for CTS without measurement of the CSA at all. Their deep learning model was trained on recognition of the nerve echogenicity and alterations in the surrounding of the nerve, but it leaves the clinician with the unease of not knowing exactly how the machine made the diagnosis.

Our results show a significant relation between weight and CSA. The correlation between body weight or BMI has been documented by others as described in Ikumi et al.^29^, who found a correlation between weight and BMI and CSA in the non-symptomatic hands of patients with unilateral CTS. More data is needed on this issue, nevertheless the body weight should be taken into account when assessing the median nerve.

While machine learning seems to be well suited to identify and measure the median nerve in its normal configuration, it still is problematic to correctly identify anatomic variations like a bifid median nerve or persistent median artery. In a well-designed study by Walker et al. the incidence for a bifid median nerve or a persistent median artery were of 8.6 and 3.7%, respectively^30^, while Granata found a frequency of 15.4% in healthy subjects and 18.5% in CTS patients^31^.

To guarantee sufficient training for machine learning, a large number of subjects with anatomical variants are needed. So far patients with bifid nerve have been excluded from studies with automated measurements^32^. In our population three healthy subjects had a bifid median nerve (5.8% of all wrists), two of them also had additional persistent median artery dividing the nerve bundle. The number of participants was too low to train the algorithm on identifying bifid nerves, so this problem still is unsolved.

**Figure 5:**
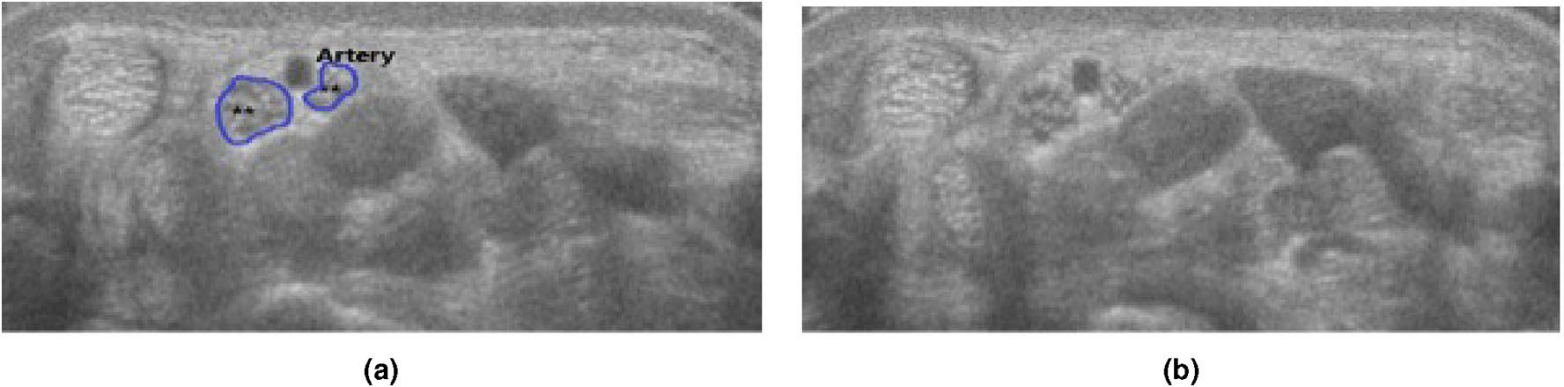
(**a**,**b**) are identical images showing the bifid nerve with the speckled pattern (blue) and between them the persistent artery.

This study has some limitations. The number of participants is relatively small and no patients or controls other than of European origin were included. Segmentation and measurement of CSA was made after recording of US, but no true blinding was established as it was possible to see from the registration chart which category (patient/control) the subject belonged to. Only the second operator I.S. involved in the inter-observer variability test was completely blinded. Limitations in the inter-observer variability: For measurement of interobserver variability both observers had to segmentate identical images that were chosen randomly. This random choosing of images was at the expense of image quality and in some images the structure of interest was difficult to be identified. Quality of segmentation: For training of the algorithm the US images were used without contrast-enhancement, which makes it more difficult to trace precisely the outline of the structures of interest, that means in real life setting the operator has an optimized image on the screen.

In conclusion the carpal tunnel inlet is a site easy to identify by visualization of the scaphoid and the pisiform bone. We believe that machine learning algorithms will evolve to a useful tool for a CSA measurement of the median nerve with high diagnostic accuracy. Automated segmentation and 3D visualization has the potential to improve the use and user experience of ultrasound and will make this imaging technique more accessible and user-friendly to a broader range of medical staff.

## Data Availability

The ultrasound datasets generated and analysed during this study, including annotations and AI model, are not publicly available due to the protocol and ethical approval of the Regional committees for medical and health research ethics in Norway (application ID 373038), but parts of the data (e.g., anonymised) could be made available from the corresponding author on reasonable request.
